# Silencing Multiple Crustacean Hyperglycaemic Hormone-Encoding Genes in the Redclaw Crayfish *Cherax quadricarinatus* Induces Faster Molt Rates with Anomalies

**DOI:** 10.3390/ijms252212314

**Published:** 2024-11-16

**Authors:** Nickolis Black, Thomas M. Banks, Susan Glendinning, Gourab Chowdhury, Donald L. Mykles, Tomer Ventura

**Affiliations:** 1 Centre for Bioinnovation, University of the Sunshine Coast, Sippy Downs, QLD 4556, Australia; 2 School of Science, Technology and Engineering, University of the Sunshine Coast, Sippy Downs, QLD 4556, Australia; 3 Department of Biology, Colorado State University, Fort Collins, CO 80523, USA; 4 Bodega Marine Laboratory, University of California, Davis, Bodega Bay, CA 94923, USA

**Keywords:** RNA interference, multigene silencing, chimeric dsRNA, gene blocks, crustaceans, decapods

## Abstract

RNA interference (RNAi)-based biotechnology has been previously implemented in decapod crustaceans. Unlike traditional RNAi methodologies that investigate single gene silencing, we employed a multigene silencing approach in decapods based on chimeric double-stranded RNA (dsRNA) molecules coined ‘gene blocks’. Two dsRNA constructs, each targeting three genes of the crustacean hyperglycaemic hormone (CHH) superfamily of neuropeptides, were produced: Type II construct targeting *Cq-*Molt-inhibiting hormone 1 (MIH1), *Cq-*MIH-like 1 (MIHL1), and *Cq-*MIHL2 isoforms and Type I construct targeting *Cq-*ion transport peptide (*Cq-*ITP; a putative hybrid of CHH and MIH) and *Cq-*CHH and *Cq-*CHH-like (CHHL) isoforms. Both constructs were injected into juvenile redclaw crayfish, *Cherax quadricarinatus*, to determine the effects of multigene knockdown on molting and developmental processes. A 20-Hydroxyecdysone (20E) enzyme-linked immunosorbent assay (ELISA) and glucose assay were used to determine the effects of RNAi on molting and hemolymph glycemic activities, respectively. Multigene silencing reduced the intermolt interval by 23%. Statistically significant elevated 20E was recorded in treated intermolt individuals, consistent with the reduced intermolt interval as well as unique and abnormal phenotypes related to the molting process, which indicates a shift in 20E-induced cascade. There was no effect of RNAi treatment on hemolymph glucose level or molt increment. Through multigene silencing and subsequent annotation of gene networks, gene blocks may provide a tailored approach to investigate complex polygenic traits with RNAi in a more efficient and scalable manner.

## 1. Introduction

### 1.1. Global Aquaculture

As the global human population is projected to reach 9.7 billion by 2050 [1], the ever-increasing demand for quality protein sources presents a significant challenge, especially in the wake of stagnating capture fishery production [2,3]. Estimates reveal that commercial fishing following its present trajectory will lead to the complete exhaustion of wild stocks within the same timeframe [4]. Emerging through necessity and innovation and offsetting demand for quality seafood, aquaculture has risen to become the world’s fastest-growing food production sector [5,6]. With a farm gate value exceeding USD 313 billion, in addition to experiencing an annual average growth rate of 3.7% (in the first three years of the 2020s), aquaculture’s contributions account for 50.9% of total seafood production [7].

### 1.2. Crustacean Aquaculture

Of the major farmed groups (fish; seaweeds and algae; molluscs; and crustaceans), crustaceans play an inimitable role on the global stage [8]. According to the Food and Agriculture Organisation’s (FAO) latest report, whilst crustacean aquaculture accounts for 13.5% of total global production, it accounts for 23% of total global export value [7]. Upon comparison with most farmed fish species, crustaceans typically fetch higher per unit values [8], and, as such, in Australia, crustacean aquaculture has sparked widespread interest at an economic level [9]. Through the development of farm dams [10], earthen ponds [10], and hatchery/grow-out operations [11], industry sectors have grown to support a variety of commercially significant species, including crayfish *Cherax destructor*, *C. albidus*, *C. cainii*, *C. tenuimanus*, *C. quadricarinatus*, spiny lobsters (*Panulirus* spp.), the black tiger prawn (*Penaeus monodon*), and the banana prawn (*Fenneropenaeus merguiensis*) [9,10,12,13,14,15].

Whilst crustacean aquaculture has experienced significant growth, both domestically and abroad, sustainability of growth trends is subject to a number of challenges, including management of environmental impacts (stemming from infrastructure development and habitat conversion) [16]; disease (bacterial, viral, and fungal infections), cannibalism, stress, and mortality in captivity [17,18,19]; calibrating desirable feed conversion efficiencies [20]; maintaining water quality parameters (dissolved oxygen, ammonia, pH, and temperature) [21,22]; sourcing desirable broodstock [23]; and the optimisation of reproductive controls to enhance breeding and reproduction [24]. In light of these challenges, a significant degree of attention has been given to the molecular mechanisms behind processes that induce spawning under controlled conditions—more specifically, the effects of and interplay between key crustacean hyperglycaemic hormone (CHH) neuropeptides that underpin commercial successes through eyestalk ablation [25].

### 1.3. Eyestalk Ablation

**Figure 1:**
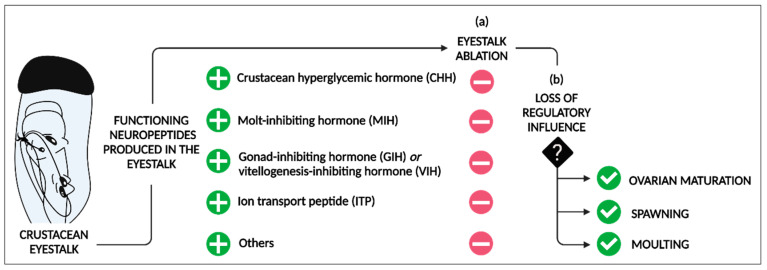
Eyestalk ablation: removal of eyestalk-produced neuropeptides (crustacean hyperglycaemic hormone (CHH), molt-inhibiting hormone (MIH), gonad-inhibiting hormone (GIH) or vitellogenesis-inhibiting hormone (VIH), ion transport peptide (ITP), and others) promotes ovarian maturation, spawning, and molting in decapods [27]. (**a**) Following ablation of the eyestalk, X-organ sinus gland-produced neuropeptides no longer exert inhibitory effects over these physiological processes, and, (**b**) in their absence, ovarian maturation, spawning, and molting ensue. Created with BioRender.com (accessed on 16 July 2024).

One controversial and ethically debated practice that continues to influence crustacean aquaculture today is eyestalk ablation. This process involves the excision of one or both eyestalks and is intended to induce spawning, ovarian maturation, and molting in decapods [26]. Located within the X-organ sinus gland complex (XO-SG), the principle neuroendocrine complex within the eyestalk, the production, storage, and release of key CHH superfamily neuropeptides tightly regulate these physiological processes [27] (Figure 1).

### 1.4. CHH Superfamily Neuropeptides

The CHH superfamily is composed of numerous pleiotropic peptides, each playing an important role in core physiological processes in decapods [27]. Recognised for their functionally diverse characteristics, CHH superfamily neuropeptides also share structural and sequence homology (signature conservation of six cysteine residues that form three intramolecular disulfide bridges); range from 72 to 80+ amino acids in size; regulate key processes of molting, reproduction, metabolism, and osmoregulation; and display tissue tropism to exert their effects [27]. Based on gene structure, the characteristics of the mature peptides, and precursor composition (the presence of a CHH-precursor-related peptide), CHH members have been classified into two distinct subgroups: type I, comprising CHH and ion transport protein (ITP), and type II, comprising molt-inhibiting hormone (MIH), gonad-inhibiting hormone (GIH)/vitellogenesis-inhibiting hormone (VIH), and mandibular organ-inhibiting hormone (MOIH) [27,28,29]. Type I members are recognised as pleiotropic hormones that direct their influence over the regulation of energetic and ionic metabolism [27,30], while type II members are more limited in function, negatively regulating processes such as molting and reproduction [27,30]. Depending on the species, CHH members may also undergo post-translational modifications, such as N-terminus cyclisation, C-terminus amidation, and F_3_ isomerisation from an L-amino acid to a D-amino acid [31]. As a result of these processes, CHH neuropeptides can exist as multiple mature isoforms within the sinus gland [31].

#### 1.4.1. Type I: Crustacean Hyperglycaemic Hormone (CHH)

CHH plays an important role across a wide range of biological processes, including the regulation of carbohydrate metabolism, lipid metabolism, reproduction, molting, osmoregulation, and metamorphosis in decapods [32,33]. Recognised largely for its roles in carbohydrate metabolism, CHH is also regarded as a pleiotropic neuropeptide, exhibiting similar activities to MIH and MOIH [27,33]. In decapods, multiple CHH genes have been identified, encoding a number of structural isoforms [27,31]. Through alternative splicing, there are two variants of CHH, a short (CHH) and long splice variant (CHH-L), which share a conserved exon toward the N-terminus but differ in sequence, slightly in length, and after the first 40 residues on the N-terminal end [27]. Unlike CHH, CHH-L does not influence hemolymph glucose levels or ecdysteroidogenesis-inhibiting activity; however, it has been shown to influence Na^+^/K^+^-ATPase and carbonic anhydrase activity [27,28]. Sharing crossover activity with type II MIH, CHH has also been implicated in the inhibition of ecdysteroid synthesis; however, its activity is less potent than MIH [27]. The mechanism of ecdysteroidogenesis inhibition is similar to that of MIH, whereby CHH is shown to maintain Y-organ (YO) basal state through G-protein-coupled receptor binding, inhibiting Rheb (Ras homolog enriched in brain)/mTOR (mechanistic target of rapamycin) activity [29].

#### 1.4.2. Type I: Ion Transport Peptide (ITP)

ITP is functionally defined for its role in stimulating the transport of Cl^−^ ions from the lumen into the hemolymph, facilitating water absorption [27]. In prawns, in addition to ionic transport, ITP’s role is suggested to extend to osmoregulation [34]. Unlike its type I counterpart CHH, ITP is yet to show any regulatory function over molting [27]. Despite recognition as a type I member [29], isoform sequencing and phylogenic analysis place ITP at the base of type I and type II [27], giving support to the creation of a third subgroup [27]. Unlike water-dwelling decapods, ITP’s role is well described in land-adapted insects, highlighting its putative roles in antidiuretic activity, adult eclosion, thirst promotion, fluid retention, and influence over phenotypic traits of cuticle melanism and wing expansion molt.

#### 1.4.3. Type II: Molt-Inhibiting Hormone (MIH)

**Figure 2:**
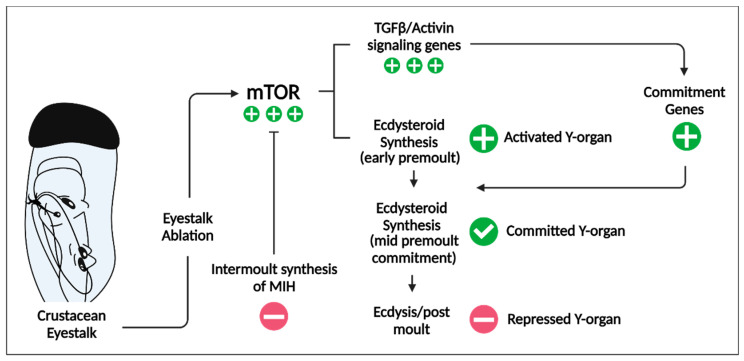
Signalling pathways that mediate Y-organ (YO) phenotype transitions throughout the molting cycle in decapods: YO basal state is preserved through cyclic nucleotide-mediated molt-inhibiting hormone (MIH) signalling, which functions to restrain mechanistic target of rapamycin (mTOR) signalling. Decreases in MIH levels (achieved through eyestalk ablation) increase mTOR activity. mTOR stimulates the synthesis of ecdysteroids, upregulating mTOR and transforming growth factor beta (TGFβ)/Activin signalling genes, simultaneously reducing the number of MIH signalling genes. Activin/myostatin signalling upregulates mTOR signalling genes and control over commitment genes that dictate the committed phenotype. Adapted from [29]. Created with BioRender.com (accessed on 16 July 2024).

MIH is an extensively studied neuropeptide, recognised for its role in inhibiting ecdysteroidgenesis in the YO [35]. Following a decrease in circulating MIH (often induced through the commercial practice of eyestalk ablation), the YO is shown to transition from a basal state to an activated one, increasing circulating titres of ecdysteroids, facilitating entry into early premolt stages (Figure 2).

The YO basal state is maintained by pulsatile releases of MIH, maintaining low levels of circulating ecdysteroid titres [36]. In between MIH pulses, the MIH signalling pathway is believed to maintain YO basal state through a triggering phase (cAMP-dependent) and a summation phase (nitric oxide/cGMP-dependent) [29]. Following YO commitment in mid-premolt stages, sensitivity to MIH is reduced but not abandoned [29]. Thus, in the event of unfavourable conditions, continued susceptibility to MIH, CHH, and other factors can still delay or suspend molt [29].

In addition to inhibition of ecdysteroidogenesis, MIH has also been implicated in the stimulation of ovarian growth by inducing vitellogenesis. In the blue crab *Callinectes sapidus*, MIH is shown to induce vitellogenin mRNA expression in the hepatopancreas and secretion during mid-vitellogenesis stages [27]. Similar observations of ovarian growth and maturation have been induced via one of two MIH isoforms (MeMIH-B and Liv-MIH2) in *Metapenaeus ensis* and *Litopenaeus vannamei*, respectively [27].

#### 1.4.4. Type II: Gonad-Inhibiting Hormone (GIH)/Vitellogenesis-Inhibiting Hormone (VIH)

GIH inhibits gonad maturation through inhibition of vitellogenin synthesis, a yolk protein precursor [37]. In *P. monodon* and *L. vannamei*, GIH-dsRNA silencing is shown to increase vitellogenin transcript levels [27]. Single GIH-dsRNA injection in *P. monodon* has also been shown to suppress GIH expression for up to 30 days, promoting ovarian maturation and spawning [27].

#### 1.4.5. Type II: Mandibular Organ-Inhibiting Hormone (MOIH)

MOIH is responsible for the mediation of vitellogenesis and reproduction in decapods [38]. MOIH functions by inhibiting the synthesis of methyl farnesoate in the mandibular organ, impacting processes of growth and reproduction that rely on this important secretory factor [27]. Following its characterisation in the crab *Cancer pagurus*, MOIH was shown to share closer sequence similarity with MIHs than CHHs [27].

### 1.5. Gene Silencing in Decapods (RNA Interference; RNAi)

RNA interference (RNAi) is a highly conserved biological mechanism in eukaryotes [39]. Utilising double-stranded RNA (dsRNA) for sequence-specific suppression of gene expression, host RNAi transpires through five steps: (1) dsRNA uptake into cells and tissues systemically, (2) Dicer-mediated cleavage of exogenous dsRNA into small interfering RNAs (siRNAs), (3) formation of an RNA-induced silencing complex (RISC) (siRNA protein complex), (4) activation of the RISC complex, and (5) subsequent mRNA degradation through siRNA-mediated target recognition and cleavage [40,41].

Applied as a molecular tool in a laboratory research setting, RNAi-based biotechnologies are well established, readily accessible, and most applicable to the field of decapod research [42,43,44]. Adopted in decapods around 2005 [41], gene silencing studies have led to advances in our understanding of functional genomics [45], host and viral immune responses [46], and systemic RNAi mechanisms [41]. In 2012, Ventura and Sagi demonstrated the first commercial use case for RNAi-based biotechnologies in decapods, successfully generating all-male monosex populations in *Macrobrachium rosenbergii* [47]. Exploiting sexual dimorphism in this species, commercial value is derived from males’ accelerated growth rates and larger sizes proportional to females [47]. Despite progress in the field of RNAi-based biotechnologies, their application in industry is subject to a number of limitations. In addition to gene silencing effects requiring efficient and scalable delivery into the organism (especially when targeting populations), repeated microinjection and electroporation of embryos is invasive and tedious, which hinders the manipulation of developmental-associated genes expressed in these early life stages [48]. The major use case of RNAi in decapods currently is for functional annotation of genes responsible for growth, development, reproduction, and metabolism, and amongst these studies, a common theme emerges: single gene silencing [41]. This approach is valuable and useful for determining gene function in many cases; however, more complex polygenic phenotypes may require the knockdown of an entire pathway or gene family to elucidate the associated molecular mechanisms [49]. While this is achievable with RNAi, often, there is significant cost and labour associated with synthesising the dsRNA substrate for multiple genes [50], which highlights the need for more efficient and high-throughput gene silencing methodologies.

### 1.6. Multigene Silencing

Multigene silencing involves the simultaneous knockdown of multiple genes using RNAi and has been demonstrated in a variety of species both in vivo and in vitro [51,52,53,54,55,56,57,58]. This approach has been explored using a range of dsRNA structures and constructs, with varying degrees of effectiveness, though, commonly, all methods aim to silence many genes while reducing the number of unique dsRNAs required [51,52,53,54,55,56,57,58]. In crustaceans, multigene silencing has been explored in the decapod species *M. rosenbergii* [59]. Unlike conventional approaches, which apply multiple dsRNAs for multiple gene targets, researchers here silenced the expression of VIH and MIH by modelling a single dsRNA spanning a shared sequence homology [59]. Beyond silencing through targeting common sequence motifs, multigene silencing in decapods remains largely unexplored in the literature.

### 1.7. Assessing the Effect of CHH Superfamily Silencing on Molt and Development in Juvenile Redclaw Crayfish (C. quadricarinatus)

**Figure 3:**
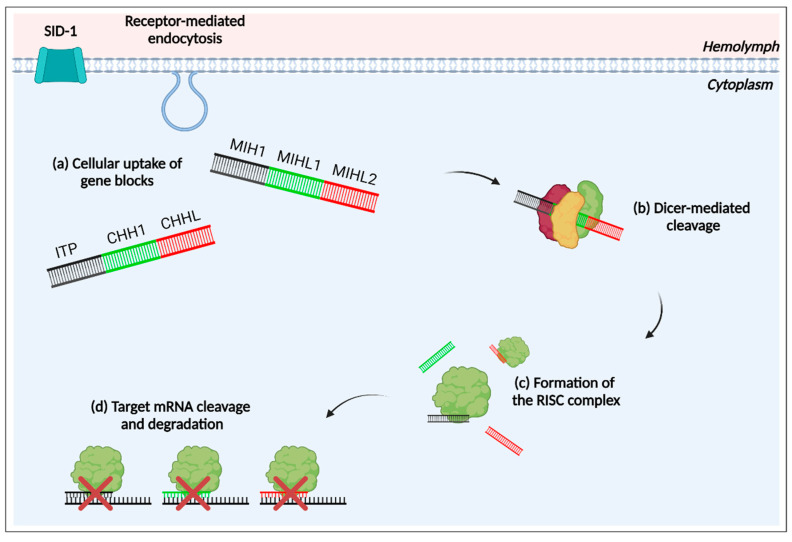
Exogenous small interfering RNA pathway (exo-siRNA): multigene silencing with gene blocks. (**a**) Systemic uptake of double-stranded RNA gene blocks (dsRNA 1: molt-inhibiting hormone 1 (MIH1); molt-inhibiting hormone-like 1 (MIHL1); and molt-inhibiting hormone-like 2 (MIHL2)) and dsRNA 2: ion transport peptide (ITP); crustacean hyperglycaemic hormone 1 (CHH1); and crustacean hyperglycaemic hormone like (CHHL) via trans-membrane dsRNA-gated channel SID-1 (systemic RNA interference deficiency) and/or receptor-mediated endocytosis [42]. (**b**) Dicer-mediated cleavage of gene blocks into small interfering RNAs (siRNA) [42]. (**c**) Resulting siRNAs are incorporated into the RNA-induced silencing complex (RISC), and an Argonaute protein within the RISC complex unwinds the duplexed siRNA, dissociating the passenger strand [42]. (**d**) The RISC complex, coupled with the remaining strand, surveys the cell for target mRNA binding through complementary base-pairing [42]. Endonucleolytic cleavage then occurs, facilitating the degradation of target mRNAs [42]. Created with BioRender.com (accessed on 16 July 2024).

A multigene silencing approach was proposed in juvenile redclaw crayfish (*C. quadricarinatus*), selected for their commercial significance in aquaculture and known host affinity to RNAi [60]. Members from type I and type II subgroups (CHH, ITP, and MIH) were selected for multigene silencing to examine their effects on molt and development in juvenile *C. quadricarinatus*. In order to test the effects of silencing, two gene block dsRNA constructs, each targeting three member peptides, were synthesised and modelled from *Cq-*CHH member peptides and their alternatively spliced counterparts: *Cq-*MIH1; *Cq-*MIHL1; *Cq-*MIHL2; *Cq-*ion transport peptide (*Cq-*ITP) (a putative hybrid of CHH and MIH) [61]; *Cq-*CHH; and *Cq-*CHHL (Figure 3).

Through synthesis and subsequent injection of gene blocks, it was hypothesised that CHH silencing would result in accelerated molting events. This multigene silencing approach has the potential to be applied to other decapod species, where single gene knock-down has proven ineffective at inducing complex and desirable phenotypes.

## 2. Results

### 2.1. Highly Efficient Silencing Observed Using dsRNA Gene Blocks

**Figure 4:**
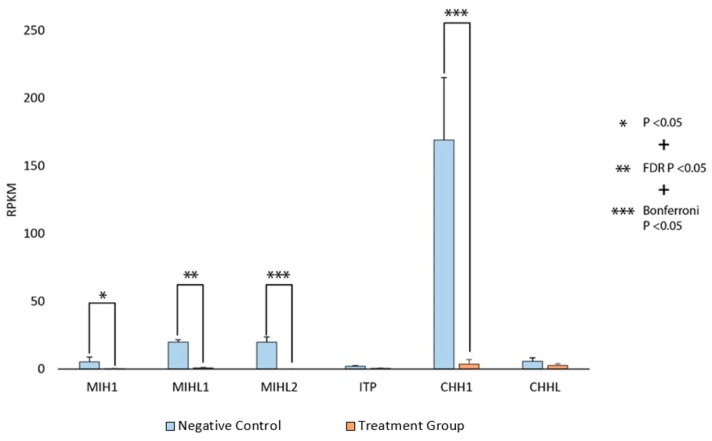
Multigene silencing of the crustacean hyperglycaemic hormone (CHH) superfamily using gene block-mediated RNA interference. Adult *Cherax quadricarinatus* males were injected with either freshwater (negative control (NC), n = 3) or gene block dsRNA (treatment group, n = 3) containing sequences complementary to the entire CHH superfamily. Expression in eyestalk ganglia was measured in reads per kilobase per million reads (RPKM), and the genes examined were molt-inhibiting hormone 1 (MIH1), molt-inhibiting hormone-like 1 (MIHL1), molt-inhibiting hormone-like 2 (MIHL2), ion transport peptide (ITP), crustacean hyperglycaemic hormone 1 (CHH1), and crustacean hyperglycaemic hormone like (CHHL) from left to right. An asterisk (*) denotes significant differential expression between the NC and treatment group in accordance with statistical analyses of increasing stringency (* *p* < 0.05, ** *p* < 0.05 and false discovery rate (FDR)-corrected *p* < 0.05, *** *p* < 0.05, FDR-corrected *p* < 0.05, and Bonferroni *p* < 0.05).

Statistical analysis of differentially expressed genes between RNA-Seq samples from eyestalks of dsRNA multigene block-silenced animals and controls revealed no significant differences. The only differentially expressed genes were members of the silenced CHH superfamily, observed one day after gene silencing. Silencing validation of the six CHH superfamily genes targeted by gene block-mediated RNAi revealed lower expression of all genes in the treatment group as opposed to the control consistently, though statistically significant silencing was only observed in four of the six targeted genes (MIH1, MIHL1, MIHL2, and CHH1, *p* < 0.05). In the case of ITP and CHHL, no significant silencing was observed, which may be a result of the low expression of these genes in the animals at the time of sampling (Figure 4).

### 2.2. No Significant Difference in Hyperglycaemic Activity and Molt Increment Between Groups

Silencing of the CHH superfamily revealed no statistically significant difference in hemolymph concentrations of glucose between the treatment group (individuals injected with gene block dsRNA) and negative control group (NC; individuals injected with freshwater. Among the treated individuals, glucose concentrations ranged from 0.24 to 0.30 mg/mL. Among the control animals, glucose concentrations ranged from 0.26 to 0.31 mg/mL (Table 1). Similarly, no statistically significant difference in molt increment was observed between groups as a result of silencing. In the treatment group, molt increment ranged between 22 and 57%. In the control animals, molt increment ranged between 20 and 60% (Table 2). There is no support for multigene silencing effects on metabolic rate (glucose metabolism and molt increment), which is controlled by type I CHHs.

### 2.3. CHH Superfamily Silencing Leads to Shorter Molt Duration and Higher Levels of Molt Hormone in Juvenile C. quadricarinatus

**Figure 5:**
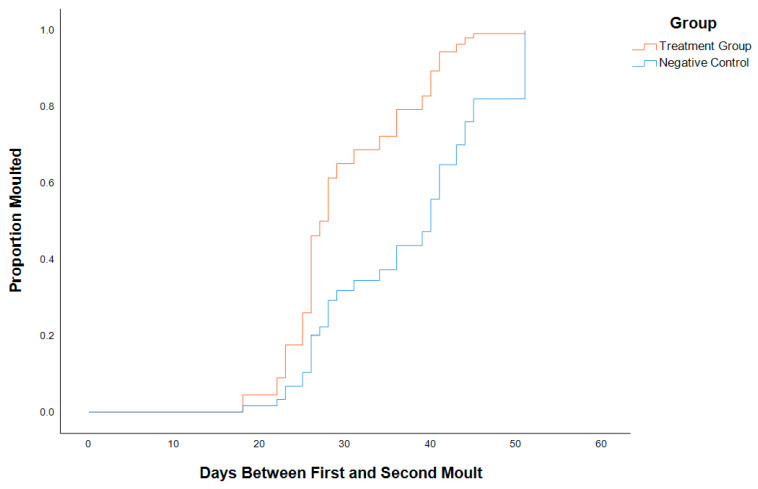
A Cox regression plot depicting the proportion of juvenile *Cherax quadricarinatus* individuals to molt against molt duration (days between first and second molt) for the treatment group (n = 17; *M* = 30.06; *SD* = 6.7682; *SE* = 1.642) and negative control group (n = 14; *M* = 37.00; *SD* = 10.968; *SE* = 2.931). An independent-samples *t*-test was statistically significant (one-tailed *p* < 0.05). Molt duration is reduced by 23.06% on average in the treatment group.

**Figure 6:**
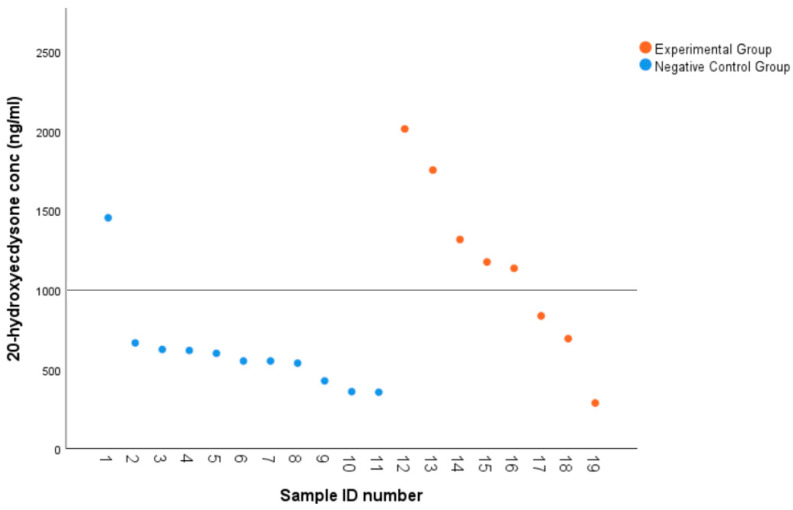
A scatterplot of intermolt juvenile *Cherax quadricarinatus* individuals and their corresponding 20-Hydroxyecdysone (20E) concentrations (ng/mL) for the treatment group (n = 8; *M* = 1152.7; *SD* = 560.2; *SE* = 198.1) and negative control group (NC) (n = 11; *M* = 613.9; *SD =* 298.4; *SE* = 90). An independent-samples *t*-test was statistically significant (one-tailed *p* < 0.05). On average, significantly lower hemolymph expression of 20E was observed in the NC as compared to the treatment group (*p* < 0.05).

CHH superfamily silencing also revealed that molt duration was significantly shorter in the treatment group compared to the negative control (*p* < 0.05). A total of 50% of animals injected with dsRNA molted before day 28 compared to the control animals, where 50% molted by day 40, and silenced individuals, on average, had a 23% reduction in molt duration compared to the control animals (Figure 5). As the CHH superfamily-silenced animals molted significantly faster than control animals, a 20-Hydroxyecdysone (20E) ELISA was performed to determine circulating levels of the molt hormone in the hemolymph of intermolt animals between treatment and control groups. Control animals showed a relatively consistent level of circulating 20E (356–667 ng/mL), with an outlier that may have been closer to molting while still in intermolt (1455 ng/mL) (Figure 6). The silenced animals, meanwhile, displayed highly variable levels of circulating 20E (288–2016 ng/mL) and, on average, had almost twice the titre of 20E in the hemolymph (*p* < 0.05) while still in the intermolt stage (Figure 6). Variations in molt duration and ecdysone concentrations give support to multigene silencing effects on molt, which are controlled by type I CHHs.

### 2.4. Abnormal Phenotypes Observed in Treatment Group Individuals Who Died During Ecdysis

#### 2.4.1. Growth of an Additional Set of Mandibles

**Figure 7:**
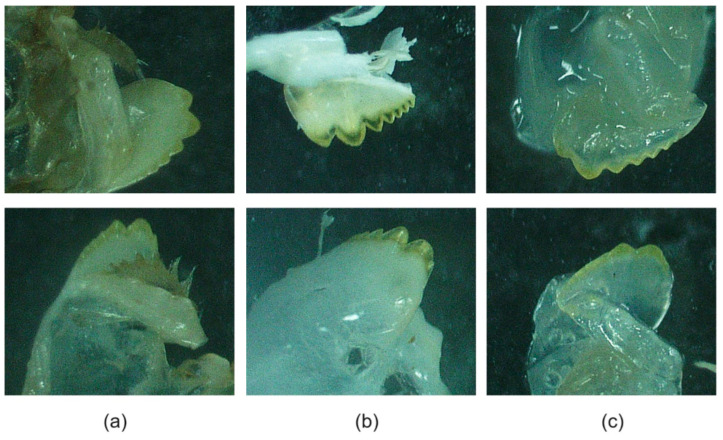
Mandible sets observed in CHH superfamily-silenced *Cherax quadricarinatus* individuals that died during ecdysis (n = 2). Animals presented with an abnormal number of mandibles, including the exuvia (**a**); a new, clean, and calcified set of mandibles (**b**); and an additional set of developing mandibles with incomplete calcification (**c**).

Two treated individuals who experienced mortality during ecdysis abnormally presented with an extra set of mandibles, where the newly forming mandibles were capped by previously formed pairs. Beneath the exuvia (Figure 7a), new, clean, and calcified mandibles have developed (Figure 7b), while the third set of partially developing mandibles presented as soft clear tissue, with no visual evidence of sclerotisation or calcification (Figure 7c). CT scans were performed to determine calcium levels of the mandible sets, which indicated that calcification did not occur in the partially developing mandibles (S1).

#### 2.4.2. Growth of an Additional Cephalothorax Cuticle

**Figure 8:**
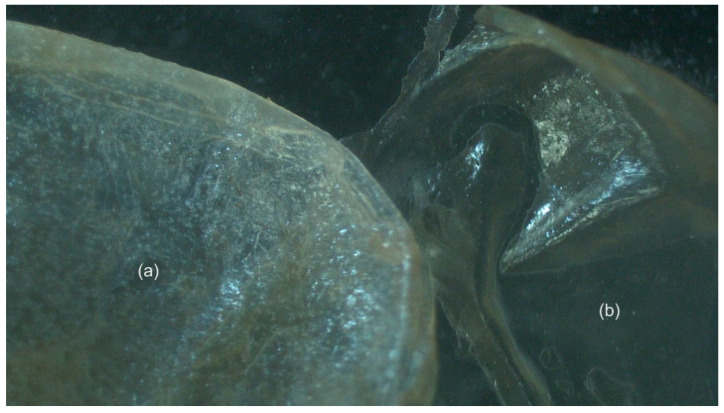
Cephalothorax cuticles observed in treated *Cherax quadricarinatus* individual that died during ecdysis (n = 1). (**a**) First cuticle. (**b**) Second cuticle.

A single individual who experienced mortality during ecdysis exhibited the growth of a second incomplete cephalothorax cuticle, newly formed underneath the exuvia (Figure 8). Upon closer examination, the first cuticle did not successfully form, experiencing only partial sclerotisation (Figure 8a). Similar to the first cuticle, the second cuticle also demonstrates aberrant formation; however, there is no visual evidence of sclerotisation, and it presents as a soft, clear tissue (Figure 8b).

## 3. Discussion

Potent silencing was observed across gene block targets. Expression levels for MIH1, MIHL1, MIHL2, and CHH1 in eyestalk ganglia transcriptomes were significantly reduced in silenced individuals. Changes in expression levels for ITP and CHHL were not statistically significant, though contained on the same gene block as CHH1 (dsRNA 2). Low baseline expression (RPKM < 10) of ITP and CHHL in the negative control group is likely to have affected statistical significance. Any effect of silencing on ITP and CHHL is less pronounced, as compared with MIH1, MIHL1, MIHL2, and CHH1. This is similar in the case of MIH1 (where the baseline expression was low), which was significantly downregulated in the treatment group but not for the FDR-corrected *p* value or Bonferroni *p* value. These data reveal that genes with naturally high expression (RPKM > 10) may be optimal for validating silencing efficiency in response to different delivery methods and dsRNA design and in species where RNAi capacity is unknown.

Individual gene blocks were designed to encompass type I and type II CHH superfamily neuropeptides. As type I neuropeptides are primarily responsible for the regulation of energetic and ionic metabolism [27,30], phenotypic effects on hemolymph glucose levels and molt increment were expected. Contrary to this prediction, type I silencing did not induce significant differences in hemolymph glucose levels between treatment and NC groups. Manfrin et al. (2015) and Li et al. (2017) also observed this phenomenon in Louisiana crawfish (*Procambarus clarkii*) when silencing CHH and associated isoforms [62,63]. Metabolic profile analysis of the muscle and hepatopancreas in *P. clarkii* following CHH silencing showed an inhibitory effect on glucose entering glycolysis in the muscle tissues, and, thus, subsequent glycogen synthesis (either intramuscularly or within the hepatopancreas) can divert glucose from the muscle back to the hepatopancreas, leading to no observable reductions as a result of intermediate hemolymph transition [63]. It should also be noted that CHH silencing effects on metabolic activity revert towards NC levels within 48 h post-injections [63]. Whilst this explanation does not explain treatment and control similarities beyond 48 h, Manfrin et al. (2015) note that the effects of CHH silencing are likely offset by other compensatory mechanisms involving other CHH superfamily neuropeptides [62]. Coinciding with results in hemolymph glucose levels, type I silencing also did not result in significant differences between groups for molt increment (% weight gain between molts). Similar to the hemolymph glucose results we observe following CHH silencing, it is likely that compensatory mechanisms arise to maintain homeostasis for crucial metabolic functions such as energy production, which may explain the lack of significant differences in molt increment between treatments. It is well established the CHH superfamily is diverse and contributes to multiple physiological pathways, and the removal of these pleiotropic neuropeptides through silencing may induce the expression of other neuropeptides to fulfil similar biological roles. This may explain why, despite CHH’s roles in energy metabolism, cessation or enhancement of growth did not occur as a result of silencing these key neuropeptides.

Giving consideration to type II neuropeptides and their intimate associations with key endocrine organs [27], we expected to significantly perturb processes of molt and development in treatment individuals. This prediction extended to the recognition of type II MIH repression characteristics over ecdysteroid production in the YO and type I CHH crossover activity in the maintenance of the YO basal state. As expected, a 23% reduction in molt latency was observed in the treatment group as compared to the NC (Figure 5). A 20E ELISA supports this with significantly higher 20E titres in the treatment group when compared to the NC (Figure 6). A comparison of 20E concentrations across molt stages (intermolt, early premolt, mid-premolt, late premolt, and post-molt) revealed elevated 20E concentrations in the treatment group for early premolt and mid-premolt stages; however, there are not enough data to draw inferences for late premolt and post-molt stages (S2). Mean 20E concentration across both groups demonstrates similar patterns to the literature across molt stages [64], peaking at the late premolt stage and declining rapidly post-molt (S3). Upon recognising high concentrations of 20E and variability across groups and molting stages, it is evident that CHH superfamily silencing imposed chronic inhibition of the negative regulatory mechanisms governing ecdysteroidogenesis. Analogous to “removing the brakes”, ecdysteroidogenesis was allowed to commence without regulation, promoting early ecdysis in the treatment group.

Similar studies have investigated the effects on phenotype following type II silencing in decapods. Pamuru et al. (2012) found a 32% reduction in molt interval following *Cq-*MIH silencing [65]; Liang et al. (2019) observed a 29% reduction in molt cycle following silencing MIH1 in *F. merguiensis* [66]; Das et al. (2015) observed higher molting rates and associated mortality in GIH silenced *P. monodon*; and Vrinda et al. (2017) noted a reduction in molt cycle following MIH1 and MIH2 silencing in *P. monodon* [67,68]. Comparatively, the decrease in molt duration observed here as a result of multigene silencing (23.06%) is lower than that described in the literature, potentially due to a difference in starting animal weight. Initial body weights for silenced individuals in Pamuru et al.’s (2012) study range from 100 to 600 mg, whilst initial body weights in the present study range from 400 to 1250 mg. The literature notes that molt frequency slows in larger individuals [69], and this may offer one explanation for the difference observed here. Additionally, variability in molt duration might have been influenced by nutrition [70], water pH [70], lighting intensity [70], water temperature [71,72], stress [73], salinity levels [70], the presence of any pollutants [74], and genetics of the population.

Silencing of type I, ITP, and CHH genes might also limit *C. quadricarinatus*’ capacity for accelerated growth. Selected for their interrelatedness [61], silencing of each may have resulted in the pleiotropic activity of other genes and/or disruption of other key regulatory processes: glycolysis; lipolysis; nicotinate and nicotinamide metabolism; amino acid synthesis; nucleotide biosynthesis; osmotic homeostasis; mediation of stress-induced hyperglycaemia; and immune regulation [27]. Despite their pleiotropy, inhibition of the peptides that govern these processes may have also impacted molt duration, which may, in part, explain the differences between single gene silencing of MIH and multigene silencing with dsRNA 1 and dsRNA 2 gene blocks.

Four treatment individuals died during ecdysis, with each individual having received at least five injections (once weekly following the first molt) (5 µg/g body weight of the dsRNA mixture). Dissection revealed the presence of two abnormal phenotypes: growth of an extra cephalothorax cuticle (sclerotisation of a single cuticle, only) (n = 1) (Figure 8) and the development of an extra set of mandibles (older mandibles capped newly formed mandible) (n = 2) (Figure 7). Physiologically, the molt cycle involves shedding of the hardened exoskeleton (exuvia), growth of a new cuticle, and hardening of said cuticle through biomineralisation processes of sclerotisation (protein–polysaccharide and protein–protein cross-linking) and calcification [75]. Under normal conditions, initiation of the molt cycle relies on cyclical pulses of ecdysone, triggering a suite of genes that coordinate tissue remodelling, cuticle release, osmotic flux, and other key steps to molt [76]. This process is managed by the ecdysone cassette (also known as ecdysone cascade) [76]. In an attempt to offer an explanation, type II silencing may have removed the sharp increase in 20E titres expected in late premolt, distributing elevated levels across early premolt, mid-premolt, and late premolt stages (S3). In response to this, elevated levels of 20E are likely triggering multiple processes of molt, in parallel, via the ecdysone cascade, downstream of the ecdysone receptor. Through multiple processes of cuticle development in parallel, incomplete cuticles may have been unable to sclerotise and mineralise following the release of an old cuticle. This may be the result of the animal not being able to store enough calcium to fully mineralise the developing cuticle and the enzymes involved in the biomineralisation not being produced at high enough levels to complement the increased rate of molting processes.

As the abnormal phenotype was only observed in two treatment individuals, further studies will need to be conducted to qualify these findings. Moreover, a thorough examination of morphology prior to treatment might paint a clearer picture of the effects of type II multigene silencing. Arguably, the injection of both constructs is comparable to eyestalk ablation. Injecting both constructs leads to the abnormalities we observed. In the blackback land crab *Gecarcinus lateralis*, eyestalk ablation induces precocious molting; like the anomalies observed in the current study, eyestalk-ablated *G. lateralis* individuals progress through premolt but fail at molt successfully [77]. In the future, injecting each construct separately would be interesting to assess whether the effect on molt can be decoupled from the effect on metabolism.

## 4. Materials and Methods

### 4.1. C. quadricarinatus Animal Rearing and Husbandry

Juvenile *C. quadricarinatus* individuals (0.44–1.25 g) were obtained from Freshwater Australian Crayfish Traders (FACT) and housed individually in submerged containers across 3 tubs within a 1600 L recirculating aquaculture system: 12 containers (0.8 L) per tub (45 L) (n = 36). Animals were maintained at 25 ± 2 °C on a 12 h light, 12 h dark cycle. Animals were fed three times per week, applying a combination of Aqua One premium-quality bloodworm (post-injection only) and tropical fish pellets (36% protein, 4% fat; Aquamunch, Maroochydore, Queensland, Australia). Adult male *C. quadricarinatus* individuals (34–55 g) were obtained from FACT and housed and maintained in communal culture (n = 6; 45 L tub) at the University of the Sunshine Coast aquaculture facilities. Animals were maintained at 24 ± 2 °C and fed twice weekly with commercially available tropical fish pellets (36% protein, 4% fat; Aquamunch, Maroochydore, Queensland, Australia).

*C. quadricarinatus* was selected for its commercial significance in aquaculture [60]. In addition, a comprehensive list of neuropeptides has been characterised in this species [61]. Juveniles were selected because they have shorter molt cycles than larger individuals [69], potential treatment effects are more pronounced in a shorter timescale, and they are easier to handle and inject frequently. A 12 h light, 12 h dark cycle for lighting was also selected to minimise stress on the animals.

### 4.2. Gene Blocks: Double-Stranded RNA Design and Production

Two double-stranded RNA (dsRNA) sequences were designed to include blocks of three 180 bp sequences per dsRNA (for a total of 540 bp per dsRNA “gene block”) derived from the CHH superfamily of neuropeptides described previously [61] (S4). The first dsRNA chimera (dsRNA 1) contained sequences derived from the MIH1, MIHL1, and MIH2 genes (S5); the second dsRNA chimera (dsRNA 2) contained sequences derived from ITP, CHH1, and CHHL genes (S5). dsRNA sequences were ordered from and produced by Genolution (Seoul, South Korea; http://genolution.co.kr/ (accessed on 1 March 2024)).

Chimeric dsRNA was designed at 540 bp for optimal cellular uptake via systemic RNA interference defective protein 1 (SID-1) transmembrane channels and receptor-mediated endocytosis [42,78,79]. The literature suggests optimal dsRNA size ranges between 150 and 750 bp in decapods [41,42]. A total of 180 bp gene sequences were selected to allow for adequate generation of siRNAs required for silencing [42].

### 4.3. Gene Silencing by RNA Interference, Dissection, RNA Extraction, and Sequencing

The two dsRNA constructs were combined in a 1:1 ratio to produce a mixture containing RNA molecules to silence all six genes of the CHH superfamily. Adult *C. quadricarinatus* individuals (35–55 g) were injected twice over a two-week period (once per week) with either 5 µg/g body weight of the dsRNA mixture (n = 6) or deionised water (n = 4) in the ventral abdominal sinus at the base of the 5th walking leg. A total of 24 h after the second injection, animals were culled on an ice slurry, and the eye stalk was dissected, harvested, and snap-frozen with liquid nitrogen for downstream RNA extraction. RNA was extracted using the RNAzol^®^ RT reagent supplemented with 1% β-mercaptoethanol as previously described and assessed via Nanodrop for quality and yield [2]. RNA from treatment (n = 3) and control (n = 3) groups was then sent to Novogene (Singapore) for paired-end sequencing using the Illumina HiSeq2500 platform (Singapore).

### 4.4. Transcriptomic Analysis and Silencing Validation

Sequencing read quality was validated using CLC Genomics Workbench 8.0.3 (CLC; Qiagen, Chadstone, VIC, Australia), low-quality nucleotides (Phred score < 20) were then removed, and the remaining high-quality reads were trimmed, removing 15 nucleotides from the 3’ end and 5 nucleotides from the 5’ end. The trimmed reads were then de novo assembled using default CLC settings with a minimum contig length of 200 bp followed by transcript quantification of each eyestalk sample as previously described [80,81]. Differential gene expression analysis was then performed and filtered based on the following parameters: fold change (absolute value) > 10, FDR corrected *p* value < 0.05, and Bonferroni *p* value < 0.05. The expression of the CHH superfamily genes used for silencing in each group was then determined to validate silencing efficiency.

Statistical analyses of increasing stringency (gene fold change (absolute value) > 10; false discovery rate (FDR)-corrected *p* value < 0.05; and Bonferroni *p* value < 0.05) were applied to reinforce the statistical significance of treatment reads that substantially deviated from NC baselines. Moreover, it highlights that non-significance is likely the result of poor baseline expression rather than impaired silencing.

### 4.5. Long-Term CHH Superfamily Silencing Trial

An in vivo silencing trial was conducted on juvenile *C. quadricarinatus* individuals (n = 36; weight range: 400–1250 mg; carapace length range: 13–20 mm) to determine the effects of chronic CHH superfamily silencing on the molting process. Following visual confirmation of the first molt (the presence of an exuvia in a housing container), individuals were sequentially assigned for injection with either deionised water (control) or 10 µg of gene block dsRNA (treatment: effectively 5 µg per gene block) at a volume of 2 µL. Animals were microinjected with a BD Ultra-Fine™ 6 mm × 31 G insulin syringe weekly in the ventral abdominal sinus at the base of the 5th walking leg 7–13 days after the first molt until each individual molted a second time. Following the second molt event, a subject’s molt duration (the time in days between the first molt and the second molt) and molt increment percentage (calculated as (1st post-molt weight–2nd post-molt weight)/(1st post-molt weight) × 100) were recorded for subsequent analysis.

Injections were scheduled weekly to accurately assess the effects of injection over time between subjects. Post-injection, individuals were fed Aqua One premium-quality bloodworm. This method was also designed to reduce animal stress and to monitor the cumulative impact of treatment over time.

### 4.6. Hemolymph Extraction and Dissection

Juvenile *C. quadricarinatus* individuals were culled on ice slurry, and hemolymph was extracted, diluted 1:1 with pre-cooled freshwater anticoagulant (140 mM NaCl, 10 mM KCl, 10 mM HEPES, 10 mM EDTA-disodium and 30 mM trisodium citrate, pH 7.3), and stored at −80 °C. Animal ID, sex (M/F/intersex), body weight (g), carapace length (mm), hepatopancreas weight (g), gastrolith status (yes/no), and molt status (intermolt; early premolt, mid-premolt, late premolt, and postmolt) were recorded.

### 4.7. 20-Hydroxyecdysone (20E) Enzyme-Linked Immunosorbent Assay (ELISA)

Hemolymph samples were defrosted and centrifuged (14,000 rpm for 10 min at 4 °C). In duplicate, 5 μL aliquots were prepared from the sample supernatant. Standard preparation and subsequent competitive ELISA were conducted according to the protocol previously described [82]. A standard curve was generated based on known 20E concentrations (49.8–2046.6 ng/mL; R^2^ = 0.938).

### 4.8. Glucose Assay

Intermolt samples without gastroliths were selected for glucose assay. Hemolymph samples were defrosted from 80 °C, centrifuged (3000 rpm for 5 min at 4 °C), and diluted 1:20 with Milli-Q water. Glucose levels were determined using the Infinity Glucose Hexokinase Liquid Stable Reagent (Thermo Fisher Scientific) according to manufacturer instructions. A standard curve was generated from known glucose concentrations (1.91–2.94 mg/mL; R^2^ = 0.999).

The rationale for selecting intermolt individuals was that they belonged to the largest molt stage grouping (n = 13). As glucose levels are known to fluctuate between molting stages [83], a comparison of glucose concentrations between intermolt individuals would best allow for statistically significant conclusions to be drawn—if any. Hemolymph samples were diluted with 1:20 Milli-Q water to bring them within the standard range.

### 4.9. Statistical Analyses

Transcriptomic analysis and silencing validation were conducted in CLC Genomics Workbench 8.0.3 (CLC; Qiagen, Chadstone, VIC, Australia) with statistical analyses of increasing stringency: *p* < 0.05, *p* < 0.05 and false discovery rate (FDR)-corrected *p* < 0.05, and *p* < 0.05, FDR-corrected *p* < 0.05, and Bonferroni *p* < 0.05. Molt duration and increment were tabulated and analysed in Microsoft Excel and IBM SPSS Statistics (version 29.0.0.0 (241)). 20E and glucose standards were generated in Microsoft Excel; sample concentrations were extrapolated from these in the same software. A Cox regression plot (one minus survival function), scatterplots, and bar charts were generated and analysed in SPSS.

## 5. Conclusions

Here, we investigated molt regulation in *C. quadricarinatus* by silencing the CHH superfamily of neuropeptides in a novel use of chimeric dsRNA-mediated RNAi. Multigene silencing of six CHH superfamily neuropeptides (MIH1; MIHL1; MIHL2; ITP; CHH1; and CHHL) significantly reduced molt latency by 23% (*p* < 0.05). As a result of CHH superfamily silencing, lethal ecdysis events were observed in four individuals, also resulting in abnormal phenotype development, including the growth of an additional cephalothorax cuticle and mandible set. Gene blocks also showcased potent silencing effects with high efficiency in vivo, demonstrating time and cost advantages over single gene silencing. Finally, this approach highlights the utility of gene blocks for large-scale application in future studies.

## Figures and Tables

**Table 1:** Hemolymph glucose levels in intermolt juvenile *Cherax quadricarinatus* across treatment and control groups.

Group	Sample Size (n)	Mean ± SEM (mg/mL)
Treatment Group	5	0.28 ± 0.02
Negative Control	8	0.29 ± 0.01

**Table 2:** Summary of molt increment (%) in juvenile *Cherax quadricarinatus* across treatment and control groups.

Group	Sample Size (n)	Mean Molt Increment ± SEM (%)
Treatment Group	16	36.51 ± 2.65
Negative Control	14	38.91 ± 3.51

## Data Availability

RNA-Seq data are available online at NCBI Sequence Read Archive with the following accession number: SAMN42566432. Additional data are contained within Supplementary Materials.

## Supplementary Materials

The following supporting information can be downloaded at: https://www.mdpi.com/article/10.3390/ijms252212314/s1.
