# Supplementary material for: Silencing Multiple Crustacean Hyperglycaemic Hormone-Encoding Genes in the Redclaw Crayfish Cherax quadricarinatus Induces Faster Molt Rates with Anomalies
Source: Int J Mol Sci. 2024 Nov 16;25(22):12314. doi: 10.3390/ijms252212314 (PMC11594818; doi:10.3390/ijms252212314)
Supplement: Supplementary file 1 [file ijms-25-12314-s001.zip › ijms-3319895-supplementary.pdf]

Supplementary Materials:

S1: CT scan of developing mandibles, gastroliths, and stomach in *Cherax quadricarinatus*

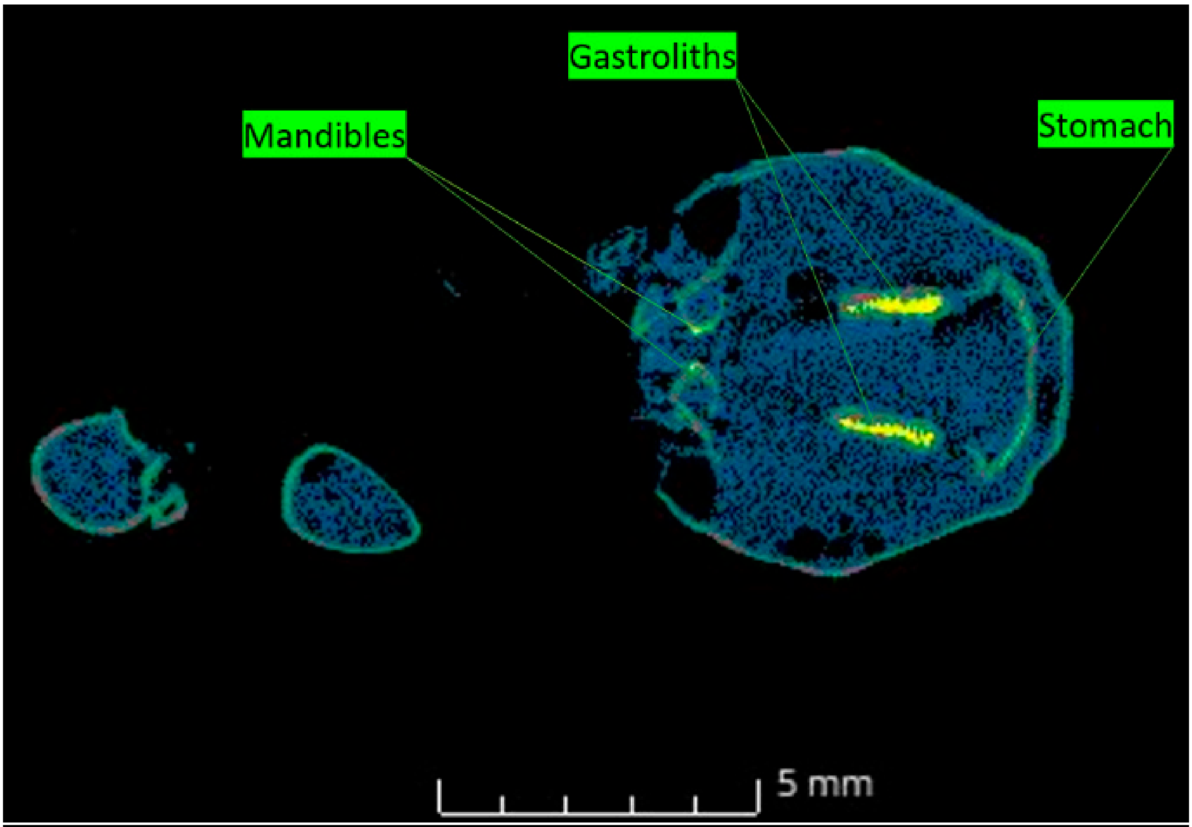

Figure S1. CT scan of mandibles, gastroliths, and stomach in treated *Cherax quadricarinatus* individual who died during ecdysis. Calcification is indicated in yellow in mandibles and gastroliths

S2: Molt metadata for silencing trial

Table S1: Molt Data of control and treated *Cherax quadricarinatus* individuals, including sample ID, molt duration, group allocation, and molt status after experiment.

**Sample ID**	**Molt Duration**	**Group**<br>**G: Treatment**<br>**A: Negative Control**	**Molt Status**
2,1	40	G	Intermolt
2,2	18	G	N/A
2,3	51	A	Intermolt
2,4	23	A	Mid premolt
2,5	99	A	Intermolt
2,6	28	G	Early premolt

2,7	45	A	Intermolt
2,8	99	A	Intermolt
2,9	26	G	Mid premolt
2,10	28	G	Mid premolt
2,11	51	A	Intermolt
2,12	39	G	Intermolt
3,1	25	A	Intermolt
3,2	22	A	Intermolt
3,3	51	A	Intermolt
3,4	26	G	N/A
3,5	36	G	Intermolt
3,6	40	A	Early premolt
3,7	41	A	Late premolt
3,8	26	G	Intermolt
3,9	28	G	N/A
3,10	43	G	Intermolt
3,11	23	G	Late premolt
3,12	26	A	Intermolt
4,1	31	A	Early premolt
4,2	41	A	Intermolt
4,3	44	A	Intermolt
4,4	27	A	Early premolt
4,5	29	G	Post molt
4,6	34	G	Intermolt
4,7	25	G	Late premolt
4,8	99	A	Mid premolt
4,9	36	G	N/A
4,10	99	G	Intermolt
4,11	26	G	Intermolt
4,12	99	A	Late premolt

S3: 20E ELISA across molt stages in *Cherax quadricarinatus*

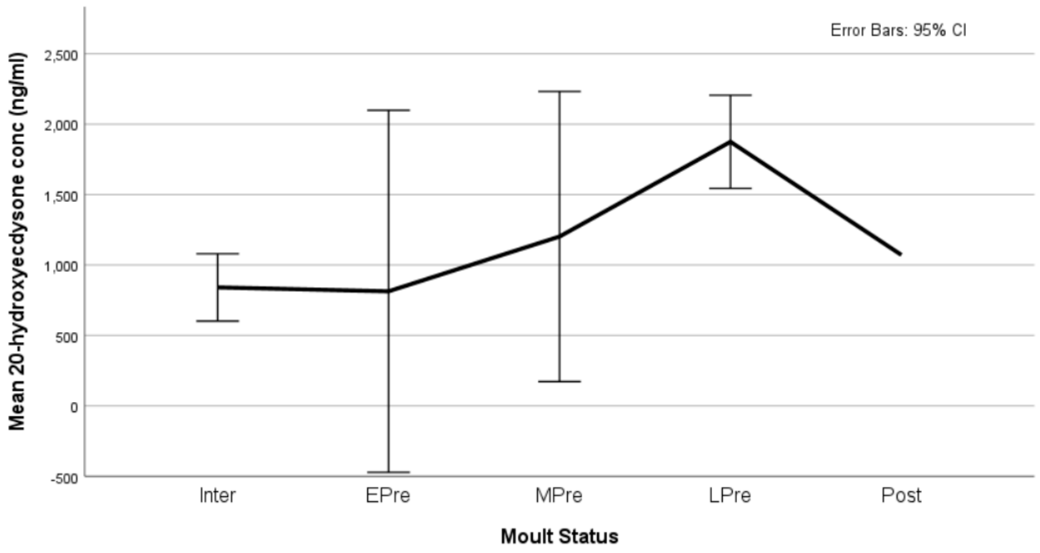

Figure S2. Mean 20E Concentration Across the Experimental Group and Control Group at Different Molt Stages

S4: CHH Superfamily Neuropeptide Sequence Data

>Cq-MIH-1

ATGGTTAAACACGCAACTCAGTCTTGCTCTGCGCTGAGGCCGTG**GCT**GATAGTGATG
GTGGTGGGTCTTCTTGTTCACCAGACAACGCCAACGTTAACGGACGACTGTCCTGGA
GCCATGGGCAACAGACATATACACACCATGTTGTTGAGAGTATGTGGAGACTGCTAT
AATGTCCTCAGAGATCCTGAAATAGAGGTTGACTGCAGGAGCGG**CTG**CTTCACCAGT
GACACATTCAAGTCGTGTCTGGAGCTTATTGAACGTGGGGATGAGTTCTTTGATTTCA
TGAGAAGGGTTGGCATTCTCAACGCTGGAGGCAAGTGA

>Cq-MIHL-1

ATGGGTAATCAGAAGTCTGAATGCTTCTACGCGCGGAGGGTGTGGCTGCTGGTGTTG
ATAGCGCTTGTTGTACAGCAGTCAACCGCACGCTTCATTGAGGACGACTGTCCCGGA
GTAGTGGGTAACAGAAACGTACACAGCATGGTGATGAGAGTATGTGAAGATTGCTAT
AACGTCTTCAGGCATCCTGAAGTAGCTGTCGGGTGTAGCGTGAGGAAGAGTACCCAG
ACTTCTTGA

>Cq-MIHL-2

ATGGGTAATCAGAAGTCTGAATGCTTCTACGCGCGGAGGGTGTGGCTGCTGGTGTTG

ATAGCGCTTGTTGTACAGCAGTCAACCGCACGCTTCATTGAGGACGACTGTCCCGGA

GTAGTGGGTAACAGAAACGTACACAGCATGGTGATGAGAGTATGTGAAGATTGCTAT

AACGTCTTCAGGCATCCTGAAGTAGCTGTCGGGTGTAGGAGGGCCTGCTTCTCCAGT

AAGATGTTCAAGTCTTGTCTCTCGGCTCTGCAGCGTGAGGAAGAGTACCCAGACTTC

TTGAGATTAATTGGCATTCTCAACGCCGGACGAAAGTGA

>Cq-ITP

ATGTCTATATTCCAGGTTTGCACACTGGGAAGGATGTGTTTGTGGTTCCTGCTTATCAT

AGGACTTCTCTCCCAGAGCCAGCAAGGTGCGGCCCATTTCTACAAGATCCAGCCTGG

CACCTTCAAAGAGTTCCAATACATCAATTGCCAAGGAACTTACAACAGGTCCACTTA

CACCAAGTTAATCCGCATCTGTGAGGAATGTCAGAACCTCTACCGCAATGACTACAC

CGTCTCGCTGGAATGCAAGGAGAACTGCTTCCAGAACGAGATGTTTGATAAATGTGT

GCTGTCTCTGCTGCTGGCTCATAAAGAAGAGGAATATAAAAACATGATTACTTATGCC

AGCGGGTAG

>Cq-CHH1

ATGACCTCCTGCAGGACGATGTGGTCACTGGTTGTGGTGGCGATGGTCGTCGTAGTG

GTCACACTGGGTCCGACTGGCGTCCGTGGACGGTCCGTGGAAGGGTCGGTGAGGCT

GGAACGACTGCTGTCGGCCGGGTCATCTTCAGCACCTCTGGGTTTCCTCTCACAAGA

TCACAGCCTCAACAAGAGACAAGTGTTCGACCAAGCTTGCAAAGGCGTCTATGACC

GAGCACTCTTCAAGAAGCTTGACCGAGTTTGTGATGATTGTTACAACCTCTATCGTAA

ACCTTACGTTGCTGCCTCCTGCAGGGAAAACTGCTACAGTAACTTGGTGTTCCGTCAG

TGTCTGGACGACCTGCTCCTTGTAGACGTTGTC ---missing --GTPCYC—

>Cq-CHHL

ATGTCACACTGTGCGGTTACGTTAGTGGTAGTGTTGGTCGTTCTAACGACGTTGTGTTC

TGGGCGTTCACTTTCTTCCGGGGAGGTTCAATGGCTTCTTCAGACGTCCCCCTACAAG

AGCTTGCCTCAAGAGTACAATGTACGAAGGAGAGATTCAGGTCTTGAATACCACAGT

GTTACTAAGAGAGCAGTCTTCGACTCACAGTGCAAAGGTTTCTACGACCGGGGCGTG

TGGGCCAGGTTGAACCGCGTCTGTGTCGACTGTCAGAATCTCTACAGATCACCTGAA

ATAGAAAACGAGTGCAGAATGGGCTGCTTCGCTACCAAATACTTCACTTCATGTGTGT

CCAACCTCCTCCTGCCTGTGGATGAGTATCAAGACATGGCTGCTTTAGTCCGTGGATC
ATAG

S5: Gene Block dsRNA Sequences for CHH Superfamily Neuropeptides

>dsRNA1; silencing sequence MIH1-MIHL1-MIHL2

GCTGATAGTGATGGTGGTGGGTCTTCTTGTTCACCAGACAACGCCAACGTTAACGGA
CGACTGTCCTGGAGCCATGGGCAACAGACATATACACACCATGTTGTTGAGAGTATG
TGGAGACTGCTATAATGTCCTCAGAGATCCTGAAATAGAGGTTGACTGCAGGAGCGG
CTGCTTCACGCTGGTGTTGATAGCGCTTGTTGTACAGCAGTCAACCGCACGCTTCATT
GAGGACGACTGTCCCGGAGTAGTGGGTAACAGAAACGTACACAGCATGGTGATGAG
AGTATGTGAAGATTGCTATAACGTCTTCAGGCATCCTGAAGTAGCTGTCGGGTGTAGC
GTGAGGAAGAGTACCCACGTACACAGCATGGTGATGAGAGTATGTGAAGATTGCTAT
AACGTCTTCAGGCATCCTGAAGTAGCTGTCGGGTGTAGGAGGGCCTGCTTCTCCAGT
AAGATGTTCAAGTCTTGTCTCTCGGCTCTGCAGCGTGAGGAAGAGTACCCAGACTTC
TTGAGATTAATTGGCATTCTCAACGC

>silencing sequence CHH-CHHL-ITP

CGGCCCATTTCTACAAGATCCAGCCTGGCACCTTCAAAGAGTTCCAATACATCAATTG
CCAAGGAACTTACAACAGGTCCACTTACACCAAGTTAATCCGCATCTGTGAGGAATG
TCAGAACCTCTACCGCAATGACTACACCGTCTCGCTGGAATGCAAGGAGAACTGCTT
CCAGAAC**GG**ACGGTCCGTGGAAGGGTCGGTGAGGCTGGAACGACTGCTGTCGGCC
GGGTCATCTTCAGCACCTCTGGGTTTCCTCTCACAAGATCACAGCCTCAACAAGAGA
CAAGTGTTCGACCAAGCTTGCAAAGGCGTCTATGACCGAGCACTCTTCAAGAAGCTT
GACCGAGTTTGTGATGATTGAGGTTCAATGGCTTCTTCAGACGTCCCCCTACAAGAGC
TTGCCTCAAGAGTACAATGTACGAAGGAGAGATTCAGGTCTTGAATACCACAGTGTT
ACTAAGAGAGCAGTCTTCGACTCACAGTGCAAAGGTTTCTACGACCGGGGCGTGTG
GGCCAGGTTGAACCGCGTCTGTGTCGAC
